# Homo Economicus Belief Inhibits Trust

**DOI:** 10.1371/journal.pone.0076671

**Published:** 2013-10-16

**Authors:** Ziqiang Xin, Guofang Liu

**Affiliations:** 1 Department of Psychology at School of Social Development, Central University of Finance and Economics, Beijing, China; 2 Institute of Developmental Psychology, Beijing Normal University, Beijing, China; Universitat Rovira i Virgili, Spain

## Abstract

As a foundational concept in economics, the homo economicus assumption regards humans as rational and self-interested actors. In contrast, trust requires individuals to believe partners’ benevolence and unselfishness. Thus, the homo economicus belief may inhibit trust. The present three experiments demonstrated that the direct exposure to homo economicus belief can weaken trust. And economic situations like profit calculation can also activate individuals’ homo economicus belief and inhibit their trust. It seems that people’s increasing homo economicus belief may serve as one cause of the worldwide decline of trust.

## Introduction

To live a happier life, people need to improve economic level as well as interpersonal trust. However, few studies have revealed a negative relationship between trust and economy. For example, in the past thirty years, Chinese economy has been rapidly growing, but that was accompanied by a dramatic decline of trust level. Concretely, Chinese college students’ interpersonal trust score decreased across birth cohorts from 82 in 1998 to 72 in 2009 on a scale with a theoretical score range from 25 to 125 [1]. The similar decline trend of trust was also revealed in other countries, such as US, UK, etc. [2], [3].

Why did trust decline rather than increase with economic bloom? One possible interpretation is that the transformation of social values could inhibit trust. In US, researchers found that the popularity of materialism was the main cause of the decline of high school students’ trust from 1976 to 1995 [4]. In China, researchers also attributed the decline of trust to the deconstruction of traditional values [1]. In our opinion, the decline of trust may be due to a more specific cause, i.e. individuals’ identification with the homo economicus belief.

### Homo Economicus Belief and Trust

Homo economicus is the most important and basic humanity hypothesis of economics, especially neoclassical economics. As the founder of economics, Smith first took self-interest as the nature of humanity in 1776 [5]. Afterwards, the concept and meanings of homo economicus have been expanded and viewed as the essential rule of human behaviors [6]. Homo economicus hypothesis assumes human behaviors are motivated by instrumental rationality and self-interest. On the one hand, individuals make decisions not intuitively and blindly, but on the basis of the deliberate judgment and calculation of costs and benefits. On the other hand, individuals are self-interested in interactions, and their sole objective is the maximization of self interest. Although late economists made some adjustments about the homo economicus assumption, these adjustments did not make major deviations from homo economicus, and which is still the most essential humanity hypothesis of economics [7].

In contrast to the humanity views of homo economicus, trust means that individuals believe their partners to take their interest into account and dare to make decisions that may be exploited [8], [9]. For instance, Mayer and colleagues suggested that trust is a willingness to take risk [9], because individuals would experience great negative consequences if their trust were exploited. In a word, we trust others, which suggests that we believe others being benevolent and not taking advantage of our trust. Thus, it seems reasonable to hypothesize that the identification with or the belief of homo economicus will destroy people’s trust to others.

Reviewing previous literature, we did not find experimental studies on the relationship between the homo economicus belief and trust. A few investigation studies have revealed negative relationships between learning economics and pro-social behaviors [e.g., 10, 11]. As for trust, only a recent study by Xin, Dou and Chen demonstrated that the experience of learning economics can depress trust [12]. They investigated the first and third grades college students’ trust, and found that economics majors showed a significant decline in trust from first to third grades, but students majoring in other humanities and social sciences did not.

In the above studies, students majoring in economics may learn and identify with the homo economicus assumption which potentially can inhibit their trust and cooperation. However, on one hand, these studies did not direct investigate the relationship between the homo economicus belief and trust; on the other hand, though Xin et al. examined the relationship [12], their study was an investigation with a cross-sectional design rather than a real experiment, which could not provide a causal interpretation. Therefore, it deserves us to conduct experiments to directly examine the destructive effect of homo economicus belief on trust.

In Experiment 1 and 2, we tested the relationship between the homo economicus belief and trust. Before trust measurement, participants in Experiment 1 were required to transcribe either an introduction of homo economicus assumption (activating the corresponding belief) or a passage about psychological methods (control condition). Participants in Experiment 2 were required to complete either two profit calculation problems (which may activate participants’ belief of homo economicus) or a general reading task (control condition). In the two experiments, we hypothesized that the activation of homo economicus belief would inhibit trust.

In Experiment 1 and 2, the transcribing of the introduction of homo economicus assumption and profit calculation problems may activate participants’ low-level construal (see the next part) rather than merely the expected belief of homo economicus. Thus, the results of Experiment 1 and 2 may be contaminated by the impact of construal level. In Experiment 3, this possibility would be eliminated.

### Construal Level Theory

Construal level theory depicts the way people mentally represent events, which was proposed by Trope and Liberman [13], [14]. They deemed that one’s representation of events can be either high- or low-level construals. High-level construal consists of general, decontextualized features and objectives that convey the abstract and essence of information about events, whereas low-level construal includes more concrete, contextual, incidental details and processes of events. Previous literature demonstrated that construal level theory can be widely used in research of consumer behaviors, judgment and decision making, etc. [15]–[17].

Construal level can be operated by different ways, such as desirability (high- level construal) and feasibility (low- level construal) [18]. Desirability reflects the superordinate and “why” aspects of actions while feasibility depicts the subordinate and “how” aspects of actions. High- and low-level construals affect human behaviors asymmetrically. For example, high-level construal can bring about stronger self control [19], and promote pro-social behaviors [20], [21].

For trust being one type of pro-social behaviors, it is reasonable to infer that construal level would affect trust too. That is, low- (high-) level construal would inhibit (promote) trust. According to the homo economicus assumption, human is rational and self-interested, and individuals make decisions on the basis of cost-benefit analysis. These characteristics were consistent with low-level construal [22]. Therefore, the transcribing of the introduction of homo economicus in Experiment 1, as well as the profit calculations in Experiment 2, may activate participants’ low-level construal. If so, the inhibition effect of homo economicus belief on trust may be actually caused by low-level construal. In Experiment 3, this possibility was eliminated.

## Experiment 1

Experiment 1 tested the hypothesis that direct contacting with the homo economicus belief would inhibit trust. Participants in the experimental condition were asked to transcribe an introduction of homo economicus, while participants in the control condition needed to transcribe a passage about psychological methods. Then, their trust was measured.

### Ethics Statement

This study (Experiment 1–3) was approved by the local ethical committee of Central University of Finance and Economics. All participants gave written informed consent.

### Participants

Sixty-two students at a Chinese university were randomly assigned into experimental (*N = *31, 16 females, *M*_age_ = 21.50 years, *SD* = 1.29) and control conditions (*N* = 31, 16 females, *M*_age_ = 21.20 years, *SD* = 2.60). Each participant got a pen as bonus.

### Materials and Procedures

Participants came into the laboratory and were informed to complete a handwriting analysis task and a social attitude test orderly. This introduction would render them not to doubt the true objective of the transcribing task. Participants in the experimental condition transcribed an introduction of homo economicus (see Appendix S1) while participants in the control condition transcribed a passage about psychological methods (see Appendix S2). These two passages both consisted of 187 Chinese characters. After the transcribing task, participants’ trust was measured by a survey of trust in others and a trust game.

The survey of trust in others was developed by Survey Research Center [23]. This survey contained three items: “General speaking, would you say that most people can be trusted, or that you cannot be too careful when dealing with people?”; “In most cases, would you say that people are helpful or self-interested?”; “If there is a chance, would you say that most people would like to take advantage of you, or fairly interact with you?”. Participants needed to make a binary response to each item. Their trust level was represented by the sum frequency of trust responses, which ranged from 0 to 3. The larger the score was, the higher participants’ trust performed.

The trust game (also named investment game) was developed by Berg, Dickhaut and McCabe [24], which has been widely used in trust measurement [25]. In the present study, the trust game was executed in survey like Buchan and Croson had done [26]. All participants decided to send X Yuan (RMB) (X = 0, 1, 2, ……, 10) to their partners and who would get 3X amount money. Then, the partners can return Y Yuan (Y = 0, 1, 2, ……, 3X) to participants. Participants’ amount sent X represents their trust level.

### Results and Discussion

**Figure 1:**
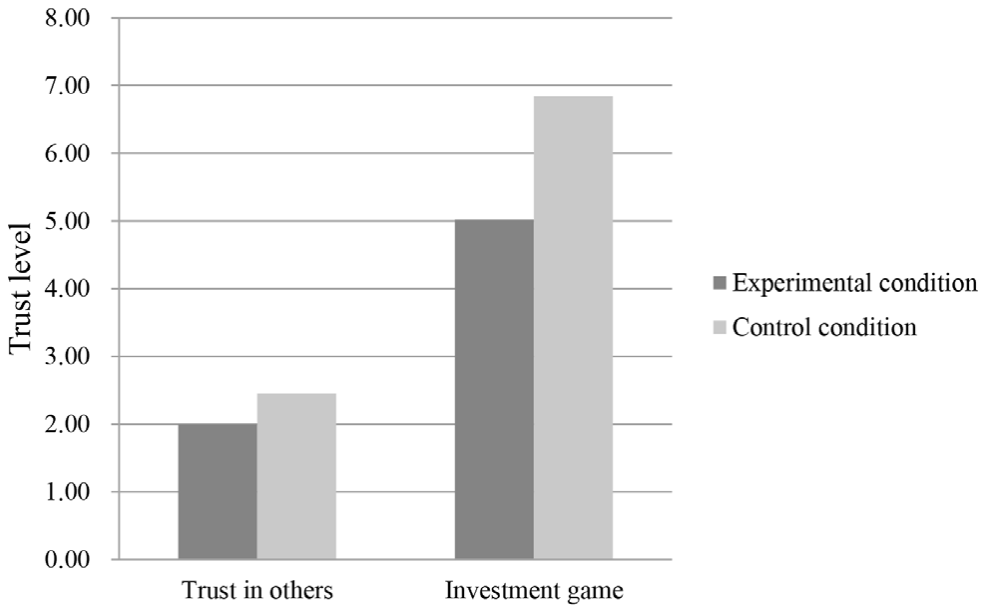
Participants’ trust level in the survey of trust in others and investment game.

In the survey, independent *t*-test showed that participants’ trust level in the experimental condition (*M* = 2.00, *SD* = 1.03) was significant lower than that in the control condition (*M* = 2.45, *SD* = 0.72), *t* (60) = 2.00, *p*<0.05, Cohen’s *d* = 0.57 (see Figure 1). In the investment game, participants’ trust level in the experiment condition (*M* = 5.02, *SD* = 2.97) was also significant lower than that in the control condition (*M* = 6.84, *SD* = 3.03), *t* (60) = 2.39, *p*<0.05, Cohen’s *d* = 0.20 (Figure 1). Moreover, participants’ trust levels measured by the survey and the trust game were significantly correlated, *r* = 0.39, *p*<0.01, indicating that these two instruments can measure the same variable. These results supported our hypothesis that direct contacting with the introduction of homo economicus activates individuals’ corresponding belief and inhibits their trust in others.

## Experiment 2

Experiment 1 demonstrated that direct contacting with the homo economicus belief can reduce trust. In daily life, however, unlike economics majors, people seldom learn the homo economicus assumption directly and explicitly, whereas, they live in economic environments such as economic interactions, profit calculations, etc., in which their belief of homo economicus may be activated. In Experiment 2, we tested whether exposure to the environment of profit calculation would lower trust.

### Participants

College student participants were randomly assigned into experimental (*N* = 32, 16 females, *M*_age_ = 22.00 years, *SD* = 1.77) and control conditions (*N = *30, 16 females, *M*_age_ = 21.26 years, *SD* = 2.15). Each participant got a pen as bonus.

### Materials and Procedures

First, participants needed to complete a priming task. In the experimental condition, participants completed two profit calculation problems (see Appendix S3), and participants in the control condition completed a reading task about the formation of loess. Second, participants’ trust was measured by the survey of trust in others as it was used in Experiment 1. Finally, to check whether the profit calculation problems activated participants’ homo economicus belief, participants evaluated their agreements of “Individuals is self- interested, they only participate in social interactions that can benefit themselves” on a 7-point scale. This item was consistent with the essence of homo economicus belief. The higher they scored on the item, the stronger they agreed with the homo economicus belief.

### Results and Discussion

We first checked whether the profit calculation problems activated participants’ homo economicus belief. In the experimental condition, participants’ mean evaluation was 4.31, *SD* = 1.55, and that in the control condition was 3.53, *SD* = 1.48. Independent *t*-test showed that participants in the experimental condition were more willing to agree with the homo economicus belief, *t* (60) = 2.02, *p*<0.05, Cohen’s *d* = 0.34. That is, the profit calculation problems successfully activated participants’ homo economicus belief.

The mean trust level of participants in the experimental condition was 2.09, *SD* = 0.86, and that in the control condition was 2.50, *SD* = 0.68. Independent *t*-test showed that participants’ trust in the experimental condition was significant lower than that in the control condition, *t* (60) = 2.06, *p*<0.05, Cohen’s *d* = 0.68. Thus, exposure to the environment of profit calculation can activate individuals’ homo economicus belief, and which can weaken trust.

## Experiment 3

As mentioned in introduction, the results of Experiment 1 and 2 may be contaminated by construal level. Experiment 3 tested this possibility by a 2 (construal level: High- versus Low- level construal)×2 (situation: Noneconomic versus Economic) design. We hypothesized that the main effect of construal level and the interaction effect between construal level and situation were all not significant, whereas situation still had a significant main effect on trust. Thus, we could determine that it was the economic situation, but not the construal level and their interaction that led to lower trust.

### Participants

One hundred and fourteen college students were randomly assigned into four conditions (see Table 1 for their demographic characteristics).

**Table 1:** Sample sizes, mean ages (years) and trust levels of participants in four conditions.

		Noneconomic situation		Economic situation	
		High-level construal	Low-level construal	High-level construal	Low-level construal
**Sample**	***N***	30 (18 females)	28 (15 females)	28 (17 females)	28 (16 females)
**Age**	***M***	18.96	19.13	19.86	20.18
	***SD***	1.24	1.78	1.78	1.54
**Trust**	***M***	2.54	2.18	2.04	2.07
	***SD***	0.58	0.86	1.00	0.98

### Materials and Procedures

Participants in the noneconomic situation read a story that Liu Ming decided to study hard to get a high academic record. Participants in the high-level construal condition needed to report 3 to 5 reasons why Liu Ming wanted a high academic record; participants in the low-level construal condition needed to propose 3 to 5 suggestions how Liu Ming could get a high academic record. In Chinese, Liu Ming is a common name, which suggests nothing special about gender, race, etc.

Participants in the economic situation were instructed to imagine that he/she was a manager of one enterprise. In the annual meeting of the corporation, he/she promised that the corporation’s annual growth rate of profit would be 50%. Participants in the high-level construal condition needed to list 3 to 5 reasons why the corporation wanted a 50% annual growth rate of profit; participants in the low-level construal condition needed to suggest 3 to 5 approaches how that growth rate could get achieved.

After completing the priming tasks, participants’ trust level was measured by the survey of trust in others same to Experiment 1.

### Results and Discussion

**Figure 2:**
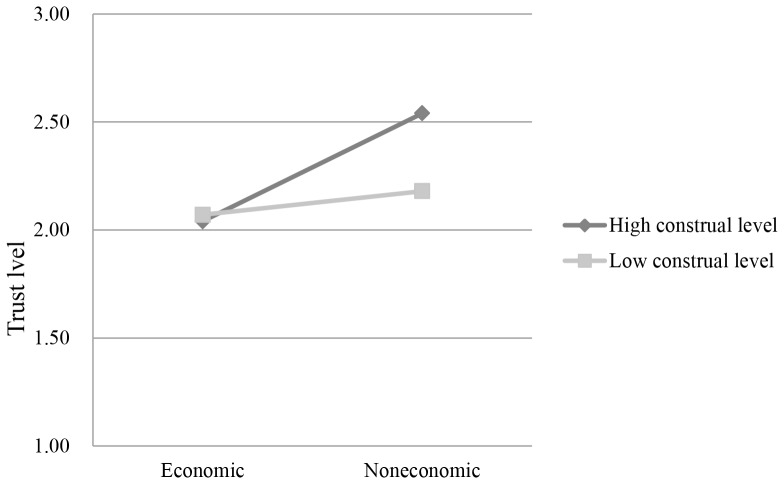
Participants’ trust level in high- and low-construal levels in economic and noneconomic situations.

The descriptions of participants’ trust level in four conditions are presented in Table 1. An analysis of variance (ANOVA) revealed that participants in the economic situation displayed a marginally significantly lower trust level (*M* = 2.05, *SD* = 0.98) than those in the noneconomic situation (*M* = 2.36, *SD* = 0.75), *F* (1, 110) = 3.49, *p* = 0.06, *η^2^* = 0.03. However, the main effect of construal level was not significant, *F* (1, 110) = 0.99, *p*>0.05, *η^2^* = 0.01, and the interaction effect between situation and construal level was not significant too, *F* (1, 110) = 1.41, *p*>0.05, *η^2^* = 0.01 (see Figure 2). That is, construal level did not affect trust but the homo economicus belief activated by the economic situation inhibited trust. It can be concluded that the results of Experiment 1 and 2 were really derived from the destructive effect of the homo economicus belief on trust, which had nothing to do with construal level.

## General Discussion

To summarize the three experiments, the present study proposed and demonstrated that the homo economicus belief can undermine trust, and individuals’ homo economicus belief can be activated by direct learning or merely exposure to economic situations, such as profit calculation, serving as an enterprise manager, etc.

Homo economicus is the most essential humanity hypothesis of economics, thus students majoring in economics may learn and identify with the homo economicus belief which emphasizes individuals are self-interested and rational. Researchers had revealed a negative relationship between economics learning and cooperation or trust [10]–[12]. These results consistently showed that there is an evident tension between the homo economicus belief and pro-social behaviors, such as trust. The present study firstly demonstrated the destructive effect of homo economicus belief on trust with the three real experiments. In Experiment 1, it is revealed that the direct and explicit contacting with homo economicus belief can reduce participants’ trust.

In daily life, however, individuals seldom contact with the assumption of homo economicus explicitly, but make cost-benefit analysis frequently in consumption, investment, as well as job selection etc. It is reasonable to believe that these situations can activate individuals’ homo economicus belief and then inhibit their trust. Experiment 2 confirmed the destructive effect of homo economicus belief on trust by exposing participants to profit calculation condition. Moreover, we eliminated the possibility that the results of Experiment 1 and 2 were derived from participants’ different construal levels.

In Experiment 3, participants in the economic situation showed a lower trust level than those in the noneconomic situation, whereas construal level did not affect trust. In Giacomantonio et al.’s view, construal level affected behaviors interacted with social motivations [27]. At the high construal level, individuals’ behaviors were more affected by their social motivation, no matter whether pro-social or pro-self. However, no significant interaction between construal level and situation was observed in our Experiment 3. We suggested that economic situations can activate people’s homo economicus belief and inhibit their trust, regardless of their construal levels.

The present study has important implications. It may shed light on the interpretation of the decline of trust in many developed and developing countries [1], [3]. With the establishment and development of market economy, people involve in economic activities more frequently than ever, which may change their humanity values and make them own a stronger belief of homo economicus, and thus inhibit their trust in other people. In a society or country, when more people participate in various economic activities, a macro decline trend in social trust may emerge from individual’s fall in trust. For example, in the past decades, China has established the system of market economy, and increasing talents with the degree of economics correlated subjects have been becoming the managers and employees of various economic departments of governments and enterprises, even common people have identified with the learned belief of homo economicus. During the process of market economy development, the decline of trust is becoming a prominent social problem. As it was found that Chinese college students’ interpersonal trust score decreased across birth cohorts from 82 in 1998 to 72 in 2009 [1], which in fact implied that society and other people were becoming untrustworthy day by day. Based on the present study, we can conclude that the learned belief of homo economicus may be a potential cause of the trust decline in the past decades in China.

We believe the similar phenomena also occur in other countries and areas. For instance, many post-communist societies (e.g. Russia) have experienced decreasing trust levels following the fall of the communist regimes, which has been regarded as the subsequence of income inequality by Bjørnskov [28]. However, the destructive effect of homo economicus belief was ignored by Bjørnskov. An earlier interview study provided indirect evidence that Russian bankers have built their self-identifications as homo economicus and the “pioneers of a money economy” during the market-style reform process in 1990s, though these identifications deviated from traditional Russian cultural-religious ideals and communist ideals [29]. Besides these bankers, we believe that common people in the societies institutionalizing a market economy have also accepted the homo economicus belief, which may serve as a destructive factor of social trust.

Finally, several future research directions should be addressed. The present study measured participants’ trust by survey and trust game. In the future study, individuals’ factual trust behaviors should be observed directly, and more economic situations (such as consumption, investment etc.) can be used to activate individuals’ homo economicus belief.

## Supporting Information

Appendix S1 (DOC)

Appendix S2 (DOC)

Appendix S3 (DOC)
