# Supplementary material for: Homo Economicus Belief Inhibits Trust
Source: PLoS One. 2013 Oct 16;8(10):e76671. doi: 10.1371/journal.pone.0076671 (PMC3797687; doi:10.1371/journal.pone.0076671)
Supplement: Appendix S1 — (DOC) [file pone.0076671.s001.doc]

***Appendix S1.***

In economics, the most important humanity hypothesis is homo economicus which indicates that human is rational and self-interested. In economic interactions, individuals’ sole objective is the maximization of self interest. For example, consumers seek satisfaction maximization, producers want profits maximization, etc.
