# Supplementary material for: Homo Economicus Belief Inhibits Trust
Source: PLoS One. 2013 Oct 16;8(10):e76671. doi: 10.1371/journal.pone.0076671 (PMC3797687; doi:10.1371/journal.pone.0076671)
Supplement: Appendix S2 — (DOC) [file pone.0076671.s002.doc]

***Appendix S2.***

In psychology, the most important method is the experiment approach which requires researchers to manipulate independent variables to investigate their effects on participants’ psychological action with the control of interferential variables. In this approach, experimental and control conditions are generally used, and reaction time, priming method are typical paradigms.
