# Supplementary material for: Homo Economicus Belief Inhibits Trust
Source: PLoS One. 2013 Oct 16;8(10):e76671. doi: 10.1371/journal.pone.0076671 (PMC3797687; doi:10.1371/journal.pone.0076671)
Supplement: Appendix S3 — (DOC) [file pone.0076671.s003.doc]

***Appendix S3.***

Problem 1:

Zhang opened a small factory. Last year, her/his factory consumed 150 000 Yuan (RMB) on raw material, and employed 25 employees with 8 000 Yuan annual salary. Finally, they produced 600 machines and each one was sold 600 Yuan.

Question: Did Zhang made a profit last year? If she/he did, how much Zhang earned?

Problem 2:

Liu decided to invest 500 000 Yuan into stock market. Finally, she/ he invested 200 000 and 300 000 Yuan in the stock of corporation X and Y respectively. Last year, Liu earned 7% in X’ stock, and lost 8% in Y’s stock.

Question: Did Liu made a profit last year? If she/he did, how much Liu earned?
